# Impact of national recommendations for routine pertussis vaccination during pregnancy on infant pertussis in Ontario, Canada: a population-based time-series study

**DOI:** 10.1186/s12884-023-05938-2

**Published:** 2023-08-31

**Authors:** Tony Antoniou, Daniel McCormack, Deshayne B. Fell, Jeffrey C. Kwong, Tara Gomes

**Affiliations:** 1 Li Ka Shing Knowledge Institute, St. Michael’s Hospital, Toronto, ON Canada; 2 ICES, Toronto, ON Canada; 3 University of Toronto, Toronto, ON Canada; 4 Department of Family and Community Medicine, St. Michael’s Hospital, Toronto, ON Canada; 5 Children’s Hospital of Eastern Ontario Research Institute, Ottawa, ON Canada; 6 School of Epidemiology and Public Health, University of Ottawa, Ottawa, ON Canada; 7 Public Health Ontario, Toronto, ON Canada

**Keywords:** Pertussis, Infant, Vaccine, Tdap, Time series analysis

## Abstract

**Background:**

In February 2018, Canada’s National Advisory Committee on Immunization (NACI) recommended antenatal tetanus–diphtheria–acellular pertussis (Tdap) immunization in every pregnancy regardless of previous Tdap immunization history. We examined the impact of the NACI recommendation on rates of infant pertussis in Ontario, Canada.

**Methods:**

We conducted a population-based time-series study of all live births in Ontario between August 1, 2011 and February 28, 2020. We used interventional autoregressive integrated moving average models to examine the impact of the NACI recommendation on monthly rates of pertussis among infants ≤ 3 months of age.

**Results:**

We observed 675 incident cases of pertussis among 1,368,024 infants 3 months of age or less between August 2011 and February 2020. The average monthly percent change in infant pertussis during the period up to and including publication of the NACI guidance and the period following publication were 0.0% (95% CI: -0.4–0.3%) and − 0.8% (95% CI -2.3% to -0.1%), respectively. Following interventional ARIMA modelling, publication of the NACI guidance was not associated with a statistically significant decrease in the monthly pertussis incidence trend (-0.67 cases per 100,000 infants; p = 0.73).

**Conclusion:**

Publication of national recommendations for antenatal Tdap immunization in every pregnancy did not significantly reduce infant pertussis rates. This may reflect the persistently low rate of antenatal vaccination following publication of the recommendations. Expanding the scope of practice of allied health care providers to include antenatal Tdap immunization and patient education regarding antenatal pertussis immunization should be considered to further optimize uptake of vaccination.

**Supplementary Information:**

The online version contains supplementary material available at 10.1186/s12884-023-05938-2.

## Introduction

Pertussis is a highly contagious respiratory tract infection associated with substantial morbidity in infants [1]. In 2019, age-specific incidence rates of pertussis reported in Canada were highest among infants less than one year of age (35.5 cases per 100,000 population) [2]. Moreover, infants under one year of age are at greatest risk of severe illness, with pertussis-related hospitalization and intensive care admission rates of 42.3 and 8.6 admissions per 100,000 population of Canadian infants, respectively, between 1999 and 2015, compared with less than one admission per 100,000 population in other age groups [3]. Importantly, the majority (76.5%) of hospitalized infants during this period were three months of age or less [3]. Similar findings have been observed in other countries, reflecting the greater vulnerability to severe pertussis among infants too young to have completed their primary three-dose immunization series against *Bordetella pertussis.* [4–8] In light of recent outbreaks, even in highly-vaccinated populations [9, 10], strategies are needed to protect young infants from pertussis and its sequelae in the period before they have initiated and/or completed their primary immunization series.

Antenatal vaccination with tetanus, diphtheria, and acellular pertussis-containing vaccine (Tdap) is an increasingly adopted strategy for preventing pertussis and its sequelae in young infants, with several countries integrating antenatal Tdap immunization into routine adult vaccination programs [11, 12]. In 2013, Canada’s National Advisory Committee on Immunization (NACI) recommended antenatal Tdap immunization during pertussis outbreaks and for pregnant women who had not been previously immunized [13]. This was updated in 2018, recommending routine antenatal Tdap immunization in every pregnancy irrespective of previous Tdap immunization history [14]. Although 27 to 32 weeks of gestation was designated as the ideal gestational timing for vaccination, thereby optimizing cord blood antibody concentrations at the time of delivery [15], NACI recommended that immunization could be undertaken at any point beyond 13 weeks of gestation [14]. These recommendations are largely aligned with those of other jurisdictions, including the United States and United Kingdom [16, 17].

A recent population-based cohort study found that antenatal Tdap vaccination among Ontario residents increased from 0.4% in 2011–2012 to 29.2% in 2019–2020 following the publication of NACI recommendations [18]. Moreover, the increase in antenatal Tdap coverage was greatest immediately following the revised guidance. However, whether the NACI guidance and the resultant increase in antenatal Tdap coverage was associated with a corresponding change in the incidence of infant pertussis remains unknown. Accordingly, we conducted a population-based time-series study to evaluate the impact of the 2018 NACI recommendations on infant pertussis incidence in Ontario, Canada.

## Methods

### Study design and setting

We conducted a population-based time-series study of all infants less than 3 months of age in Ontario between August 1, 2011 and February 28, 2020. We selected this time period to allow a sufficiently long observation period prior to the publication of the NACI guidance and to avoid the confounding effects of COVID on the incidence and diagnosis of other respiratory infections. Ontario residents have universal access to hospital care and physicians’ services, including prenatal care. However, Ontario did not have a publicly-funded program for repeated Tdap vaccination, including during pregnancy, in place during the study period.

### Data sources and study population

We used Ontario’s administrative health databases, which are securely linked using unique, encoded identifiers and analyzed at ICES in Toronto, Ontario (https://www.ices.on.ca). We used the Canadian Institute for Health Information National Ambulatory Care Reporting System and Discharge Abstract Database to identify pertussis-related emergency department visits and hospital admissions, respectively, among infants 3 months of age or lower. These databases contain detailed clinical information regarding all emergency department visits and hospital admissions in Ontario. We used the Ontario Health Insurance Plan (OHIP) database to identify claims for physician services, including immunizations identified through fee codes, and obtained basic demographic data from the Registered Persons Database, a registry of all Ontario residents eligible for health insurance. The use of data in this project is authorized under section 45 of Ontario’s Personal Health Information Protection Act, which does not require review by a Research Ethics Board.

### Study outcomes

Our primary outcome was the monthly incidence of pertussis in infants 3 months of age or less. We defined an incident case as an outpatient physician visit [International Classification of Diseases, Ninth Revision (ICD-9), code 033] or an emergency department visit or hospital admission [International Classification of Disease, 10th Revision (ICD-10), code A37] with a diagnosis of pertussis. To prevent misclassification due to outpatient visits for infant pertussis vaccination, we excluded all infant physician encounters that included OHIP fee codes associated with any pertussis-containing immunizations (G840, G841, G847).

### Statistical analysis

We determined the crude average monthly percent change prior to and following the NACI guidance. Because the average monthly percent change does not account for prior trends, temporal correlation of the time series and seasonality, we used interventional autoregressive integrated moving average (ARIMA) models to estimate the association between the NACI guidance and infant pertussis rates [19, 20]. We used the Dickey Fuller test to determine the stationarity of the time series, and applied first order and seasonal differencing to arrive at a stationary series [20, 21]. We used the autocorrelation function and partial autocorrelation function to identify autoregressive and/or moving average components in the time series and correct for autocorrelation remaining after differencing, and selected the best models using goodness-of-fit tests [20, 21]. We used residual plots and the Portmanteau statistic to confirm that residuals from specified ARIMA models were a white noise process [19, 20, 22]. Finally, once the ARIMA models were specified, we used a ramp intervention function to test for a gradual slope change in antenatal Tdap immunization beginning one month following the NACI guidance (i.e., March 2018) [20].

We conducted several analyses to triangulate and test the robustness of our findings. First, we used ordinary least squares segmented regression with Newey–West standard errors to account for heteroscedasticity and with *k* lags to account for autocorrelation, where the value for *k* was determined using the Cumby-Huizinga general test for autocorrelation [23, 24]. Next, we conducted a structural break analysis of each time series by plotting the cumulative sums of ordinary least squares residuals (CUSUM-OLS) and corresponding confidence bands over the study period [25]. A structural break is identified by movement of the plotted CUSUM beyond the confidence bands and the associated test statistic. All analyses were completed using SAS Enterprise Guide, version 7.1 (SAS Institute Inc., Cary, NC, USA), R Studio, and Stata version 17.0 (Stata Corp LP, TX).

## Results

**Fig. 1:**
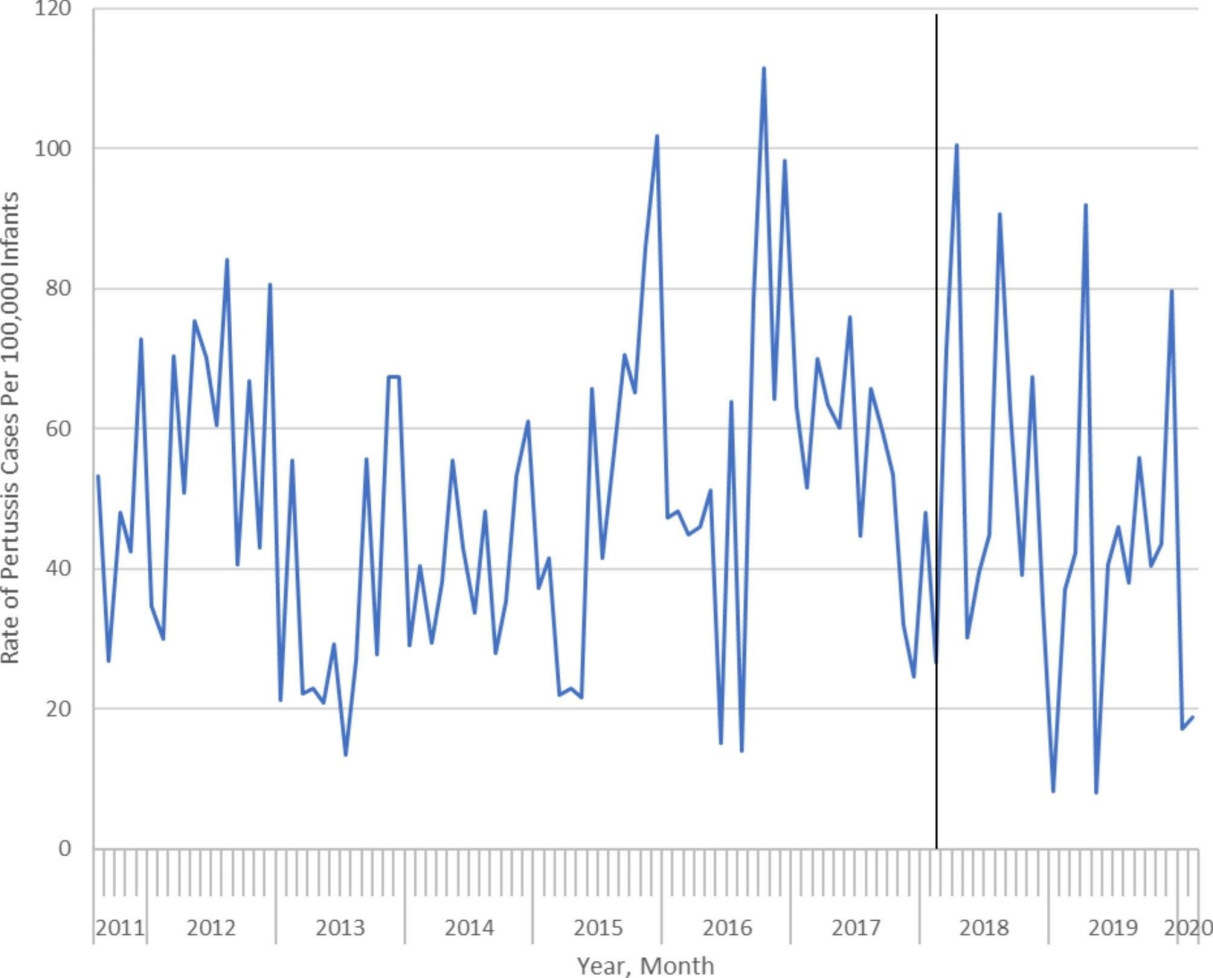
Monthly rate of incident infant pertussis per 100,000 infants ≤ 3 months of age in Ontario, Canada between August 2011 and February 2020

There were 675 incident cases of pertussis among 1,368,024 infants 3 months of age or less between August 2011 and February 2020, with 121 (17.9%), 288 (42.7%) and 266 (39.4%) being diagnosed between the ages of 0 to 1 months, 1 to 2 months and 2 to 3 months, respectively. Most infants (n = 475; 70.4%) were diagnosed as outpatients, while 76 (11.3%) and 124 (18.4%) cases were diagnosed in the emergency department and during an inpatient hospitalization, respectively. In total, 189 infants were hospitalized with pertussis, of whom 49 (25.9%) required admission to an intensive care unit (ICU). Monthly rates of infant pertussis were 53.2 cases per 100,000 population and 18.8 cases per 100,000 population in August 2011 and February 2020, respectively (Fig. 1). The average monthly percent change (AMPC) in incident pertussis over the entire study period was − 0.2% (95% CI -0.7–0.1%). The AMPCs during the period up to and including publication of the NACI guidance and the period following publication were 0.0% (95% CI: -0.4–0.3%) and − 0.8% (95% CI -2.3% to -0.1%), respectively.

In interventional ARIMA modelling, publication of the NACI guidance was not associated with a statistically significant decrease in the monthly pertussis incidence trend (-0.67 cases per 100,000 infants; p = 0.73). We also observed similar findings in sensitivity analyses, with no structural breaks identified using the OLS-CUSUM test (Supplemental Appendix 1) and a non-significant decrease in the monthly pertussis incidence trend of -1.2 cases per 100,000 infants (95% CI: -2.5 to 0.2 cases per 100,000 infants) following NACI guidance publication using segmented regression.

## Discussion

In our population-based time-series study, we found a crude reduction in pertussis incidence among young infants over time. However, in interrupted time series models that accounted for trend and seasonality, the publication of the NACI guidance did not specifically influence these trends. Our findings likely reflect persisting low antenatal Tdap coverage in Ontario during the study period, with past research finding that immunization was undertaken in approximately one in four pregnancies in the 12 months following publication of the NACI guidance [18].

Our study has several implications for public health. Antenatal pertussis immunization is an effective intervention for mitigating the burden of severe pertussis illness in young infants, with estimates of vaccine effectiveness ranging from 36 to 90% in several studies [26–34]. Further, antenatal Tdap immunization is safe, with multiple studies finding no association between vaccination and adverse fetal or neonatal outcomes [12, 34–37]. Yet, despite supportive evidence, several studies have documented suboptimal uptake of maternal pertussis immunization [38–42]. Because healthcare provider recommendation is a key determinant of antenatal Tdap immunization [43, 44], reluctance of maternity care providers to administer vaccines during pregnancy in the absence of local recommendations or endorsement of a professional body is a potentially important reason for suboptimal uptake. This assertion is supported by prior research demonstrating that only 35% of surveyed Canadian maternity care providers routinely recommended pertussis vaccination during pregnancy in the period predating the publication of the updated NACI recommendations [45]. Insufficient training and concerns about vaccine safety are among the principal reasons provided by healthcare providers for not recommending antenatal Tdap immunization [45, 46]. Specifically, a cross-sectional web-based survey of Canadian maternity providers found that relative to physicians and nurses, a lower proportion of midwives and pharmacists considered Tdap to be an effective means for protecting infants [45]. Moreover, midwives had greater reservations about the safety of Tdap during pregnancy, whereas pharmacists were more likely to report that other maternity care providers discuss vaccination with their pregnant patients [45]. Although inferences from this study are limited by its non-representative sample and the possibility that perspectives have changed since its publication in 2020, these findings suggest that greater training and education regarding vaccine safety and efficacy, as well as expanding the scope of pharmacy practice to include Tdap, may increase maternity provider endorsement of this measure.

In addition, funding for repeated Tdap vaccination varies across Canada, and the lack of a publicly funded program for repeated Tdap immunization in Ontario during the study period may have limited uptake of vaccination during pregnancy. However, a national survey of 4,607 mothers conducted following the publication of NACI guidance found that cost was cited as the reason for non-vaccination by less than 1% of unvaccinated mothers, with lack of awareness about pertussis immunization being the main reason for not having been vaccinated [47]. Although the province of Ontario expanded its publicly funded Tdap immunization program in April 2022 to include a routine dose in every pregnancy [48], additional strategies may be needed to increase awareness and maximize coverage of Tdap immunization among pregnant women. Example interventions that have been demonstrated to be effective include providing education to increase provider awareness of vaccine recommendations during pregnancy and expanding the scope of midwives and pharmacists to include antenatal Tdap immunization [49, 50]. Although evidence-based, these strategies require policy change and the involvement of professional regulatory bodies for implementation.

In addition to persistently low rates of antenatal Tdap vaccination, our findings may reflect a preponderance of milder illness among infants during our study period, with only 28.0% of cases requiring hospitalization. Because antenatal Tdap vaccination appears less effective at preventing mild illness (i.e., illness not leading to hospital admission) [31], the large number of infants diagnosed as outpatients may have obfuscated the clinical impact of increased maternal vaccination rates following the NACI guidance.

Our study has some limitations. First, the validity of the diagnostic codes used to define cases of infant pertussis is unknown. However, rates of infant pertussis derived in our study approximated published estimates of laboratory-confirmed cases [2]. Second, the very low rate of infant pertussis and inherent variability in the occurrence of this outcome may have resulted in a lack of power for detecting a significant change following the publication of NACI guidance and also precluded our ability to evaluate just severe pertussis (i.e., illness leading to hospital admission) [51]. For similar reasons, we could not replicate our analyses stratified by age group. Third, as with all interrupted time series studies, temporal confounding related to discrete events occurring in close proximity to the interventions of interest or changes in the source population is a potential source of bias. However, we are unaware of any co-occurring interventions, and there were no other interventions implemented during this period that could confound our findings.

In summary, we found no appreciable change in infant pertussis rates in Ontario following the publication of the NACI immunization standard recommending antenatal Tdap immunization in all pregnancies. This finding most likely reflects persistently suboptimal antenatal Tdap coverage. Further research is needed to understand how health care providers interpret and promote NACI guidance to their patients. In addition, expanding the scope of allied health professionals such as midwives and pharmacists to include Tdap immunization and programs to promote awareness of the updated guidance among clinicians and patients should be considered to further increase uptake of antenatal Tdap immunization. Moreover, research exploring uptake of evidence regarding the effectiveness of vaccination during pregnancy and expanding the pool of health care professionals able to provide vaccines to pregnancy is also relevant to other vaccines recommended during the antenatal period, including influenza and COVID-19 [52]. The latter recommendation is especially important in light of recent evidence confirming a reduced risk of severe illness and death among women with complete or boosted vaccine doses during the Omicron phase of the COVID-19 pandemic [53].

### Electronic supplementary material

Below is the link to the electronic supplementary material.

Supplementary Material 1

## Data Availability

The data set from this study is not available publicly and is held securely in coded form at ICES. While data sharing agreements prohibit ICES from making the data set publicly available, access may be granted to those who meet pre-specified criteria for confidential access, available at www.ices.on.ca/DAS (email: das@ices.on.ca). The full data set creation plan and underlying analytic code are available from the authors upon request (tony.antoniou@unityhealth.to), understanding that the programs may rely upon coding templates or macros that are unique to ICES.

## List of abbreviations

AMPC: Average Monthly Percent Change
ARIMA: Autoregressive Integrated Moving average
CUSUM-OLS: Cumulative sums of ordinary least squares
ICD-10: International Classification of Disease, 10th Revision
ICD-9: International Classification of Diseases, Ninth Revision
NACI: National Advisory Committee on Immunization
OHIP: Ontario Health Insurance Plan
Tdap: Tetanus, Diphtheria, and Acellular Pertussis-containing vaccine

## References

[1] Greenberg DP, von König CH, Heininger U (2005). Health burden of pertussis in infants and children. Pediatr Infect Dis J.

[2] Public Health Agency of Canada. Notifiable diseases online. https://dsol-smed.phac-aspc.gc.ca/notifiable/charts?c=cc. 2022.

[3] Abu-Raya B, Bettinger JA, Vanderkooi OG, Vaudry W, Halperin SA, Sadarangani M (2020). Members of the canadian immunization monitoring program, active (IMPACT). Burden of children hospitalized with pertussis in Canada in the Acellular Pertussis Vaccine Era, 1999–2015. J Pediatr Infect Dis Soc.

[4] Zumstein J, Heininger U, Swiss Paediatric Surveillance Unit (SPSU) (2021). Decline of pertussis in hospitalised children following the introduction of immunisation in pregnancy - results from a nationwide, prospective surveillance study, 2013–2020. Swiss Med Wkly.

[5] Carlsson RM, von Segebaden K, Bergstrom J, Kling AM, Nilsson L (2015). Surveillance of infant pertussis in Sweden 1998–2012; severity of disease in relation to the national vaccination programme. Euro Surveill.

[6] Mbayei SA, Faulkner A, Miner C, Edge K, Cruz V, Peña SA (2019). Severe pertussis infections in the United States, 2011–2015. Clin Infect Dis.

[7] Gabutti G, Rota MC, Bonato B, Pirani R, Turlà G, Cucchi A (2012). Hospitalizations for pertussis in Italy, 1999–2009: analysis of the hospital discharge database. Eur J Pediatr.

[8] Chang IF, Lee PI, Lu CY, Chen JM, Huang LM, Chang LY (2019). Resurgence of pertussis in Taiwan during 2009–2015 and its impact on infants. J Microbiol Immunol Infect.

[9] Billingsley M (2012). Pregnant women in UK are offered whooping cough vaccine to protect newborns. BMJ.

[10] Cherry JD (2012). Epidemic pertussis in 2012–the resurgence of a vaccine-preventable disease. N Engl J Med.

[11] Switzer C, D’Heilly C, Macina D (2019). Immunological and clinical benefits of maternal immunization against Pertussis: a systematic review. Infect Dis Ther.

[12] Gkentzi D, Katsakiori P, Marangos M, Hsia Y, Amirthalingam G, Heath PT (2017). Maternal vaccination against pertussis: a systematic review of the recent literature. Arch Dis Child Fetal Neonatal Ed.

[13] National Advisory Committee on Immunization. An Advisory Committee Statement (ACS) National Advisory Committee on Immunization (NACI). Update on pertussis vaccination in pregnancy. https://www.canada.ca/en/public-health/services/publications/healthy-living/update-pertussis-vaccination-pregnancy.html. 2022.

[14] National Advisory Committee on Immunization. An Advisory Committee Statement (ACS) National Advisory Committee on Immunization (NACI). update on immunization in pregnancy with tetanus toxoid, reduced diphtheria toxoid and reduced acellular pertussis (Tdap) vaccine. https://www.canada.ca/en/public-health/services/publications/healthy-living/update-immunization-pregnancy-tdap-vaccine.html. 2022.

[15] Naidu MA, Muljadi R, Davies-Tuck ML, Wallace EM, Giles ML (2016). The optimal gestation for pertussis vaccination during pregnancy: a prospective cohort study. Am J Obstet Gynecol.

[16] Centers for Disease Control and Prevention (2013). Updated recommendations for use of tetanus toxoid, reduced diphtheria toxoid, and acellular pertussis vaccine (Tdap) in pregnant women—Advisory Committee on Immunization Practices (ACIP), 2012. MMWR Morb Mortal Wkly Rep.

[17] UK Health Security Agency. Pertussis vaccination programme for pregnant women update: vaccine coverage in England, July to September 2021. https://assets.publishing.service.gov.uk/government/uploads/system/uploads/attachment_data/file/1056185/FINAL-HPR0322-PRTSSS-vc-Q2_21022022.pdf. 2022.

[18] Fakhraei R, Fung S, Petrcich W, Crowcroft N, Bolotin S, Gaudet L (2022). Trends and characteristics of tdap immunization during pregnancy in Ontario, Canada: a retrospective cohort study. CMAJ Open.

[19] Helfenstein U (1991). The use of transfer function models, intervention analysis and related time series methods in epidemiology. Int J Epidemiol.

[20] Schaffer AL, Dobbins TA, Pearson SA (2021). Interrupted time series analysis using autoregressive integrated moving average (ARIMA) models: a guide for evaluating large-scale health interventions. BMC Med Res Methodol.

[21] Dickey DA, Fuller WA (1979). Distribution of the estimators for autoregressive time series with a unit root. J Am Stat Assoc.

[22] Ljung GM, Box GEP (1978). On a measure of lack of fit in time series models. Biometrika.

[23] Newey WK, West KD (1987). A simple, positive semi-definite, heteroskedasticity and autocorrelation consistent covariance matrix. Econometrica.

[24] Cumby RE, Huizinga J (1992). Testing the autocorrelation structure of disturbances in ordinary least squares and instrumental variables regression. Econometrica.

[25] Ploberger W, Krämer W (1992). The CUSUM test with OLS residuals. Econometrica.

[26] Becker-Dreps S, Butler AM, McGrath LJ, Boggess KA, Weber DJ, Li D (2018). Effectiveness of prenatal tetanus, diphtheria, acellular pertussis vaccination in the prevention of infant pertussis in the U.S. Am J Prev Med.

[27] Winter K, Nickell S, Powell M, Harriman K (2017). Effectiveness of prenatal versus postpartum tetanus, diphtheria, and acellular pertussis vaccination in preventing infant pertussis. Clin Infect Dis.

[28] Dabrera G, Amirthalingam G, Andrews N, Campbell H, Ribeiro S, Kara E (2015). A case-control study to estimate the effectiveness of maternal pertussis vaccination in protecting newborn infants in England and Wales, 2012–2013. Clin Infect Dis.

[29] Amirthalingam G, Andrews N, Campbell H, Ribeiro S, Kara E, Donegan K (2014). Effectiveness of maternal pertussis vaccination in England: an observational study. Lancet.

[30] Baxter R, Bartlett J, Fireman B, Lewis E, Klein NP (2017). Effectiveness of vaccination during pregnancy to prevent infant pertussis. Pediatrics.

[31] Saul N, Wang K, Bag S, Baldwin H, Alexander K, Chandra M (2018). Effectiveness of maternal pertussis vaccination in preventing infection and disease in infants: the NSW Public Health Network case-control study. Vaccine.

[32] Skoff TH, Blain AE, Watt J, Scherzinger K, McMahon M, Zansky SM (2017). Impact of the US maternal tetanus, Diphtheria, and Acellular Pertussis Vaccination Program on preventing Pertussis in Infants < 2 months of age: a case-control evaluation. Clin Infect Dis.

[33] Bellido-Blasco J, Guiral-Rodrigo S, Míguez-Santiyán A, Salazar-Cifre A, González-Morán F (2017). A case-control study to assess the effectiveness of pertussis vaccination during pregnancy on newborns, Valencian Community, Spain, 1 March 2015 to 29 February 2016. Eurosurveillance.

[34] Vygen-Bonnet S, Hellenbrand W, Garbe E, von Kries R, Bogdan C, Heininger U (2020). Safety and effectiveness of acellular pertussis vaccination during pregnancy: a systematic review. BMC Infect Dis.

[35] McMillan M, Clarke M, Parrella A, Fell DB, Amirthalingam G, Marshall HS (2017). Safety of Tetanus, Diphtheria, and Pertussis Vaccination during pregnancy: a systematic review. Obstet Gynecol.

[36] Fakhraei R, Crowcroft N, Bolotin S, Sucha E, Hawken S, Wilson K (2021). Obstetric and perinatal health outcomes after pertussis vaccination during pregnancy in Ontario, Canada: a retrospective cohort study. CMAJ Open.

[37] Laverty M, Crowcroft N, Bolotin S, Hawken S, Wilson K, Amirthalingam G (2021). Health Outcomes in Young Children following pertussis vaccination during pregnancy. Pediatrics.

[38] Maertens K, Braeckman T, Blaizot S, Theeten H, Roelants M, Hoppenbrouwers K (2018). Coverage of recommended vaccines during pregnancy in Flanders, Belgium. Fairly good but can we do better?. Vaccine.

[39] Laenen J, Roelants M, Devlieger R, Vandermeulen C (2015). Influenza and pertussis vaccination coverage in pregnant women. Vaccine.

[40] Deverall EJ, Gilmore B, Illing S, Peiris-John R (2018). Pertussis vaccination uptake in pregnancy: lessons to be learned from an integrated healthcare approach. N Z Med J.

[41] Bödeker B, Walter D, Reiter S, Wichmann O (2014). Cross-sectional study on factors associated with influenza vaccine uptake and pertussis vaccination status among pregnant women in Germany. Vaccine.

[42] Quattrocchi A, Mereckiene J, Fitzgerald M, Cotter S (2019). Determinants of influenza and pertussis vaccine uptake in pregnant women in Ireland: a cross-sectional survey in 2017/18 influenza season. Vaccine.

[43] McQuaid F, Jones C, Stevens Z, Plumb J, Hughes R, Bedford H (2016). Factors influencing women’s attitudes towards antenatal vaccines, group B streptococcus and clinical trial participation in pregnancy: an online survey. BMJ Open.

[44] Varan AK, Esteves-Jaramillo A, Richardson V, Esparza-Aguilar M, Cervantes-Powell P, Omer SB (2014). Intention to accept Bordetella pertussis booster vaccine during pregnancy in Mexico City. Vaccine.

[45] Dubé E, Gagnon D, Kaminsky K, Green CR, Ouakki M, Bettinger JA (2020). Vaccination during pregnancy: canadian maternity care providers’ opinions and practices. Hum Vaccin Immunother.

[46] Bonville CA, Cibula DA, Domachowske JB, Suryadevara M (2015). Vaccine attitudes and practices among obstetric providers in New York State following the recommendation for pertussis vaccination during pregnancy. Hum Vaccin Immunother.

[47] Gilbert NL, Guay M, Kokaua J, Lévesque I, Castillo E, Poliquin V (2022). Pertussis vaccination in canadian pregnant women, 2018–2019. J Obstet Gynaecol Can.

[48] Ontario Ministry of Health. Tdap (tetanus, diphtheria, pertussis) vaccine program. https://www.health.gov.on.ca/en/public/programs/immunization/docs/tdap_fs_en.pdf. 2022.

[49] Mohammed H, McMillan M, Roberts CT, Marshall HS (2019). A systematic review of interventions to improve uptake of pertussis vaccination in pregnancy. PLoS ONE.

[50] Howe AS, Gauld NJ, Cavadino AY, Petousis-Harris H, Dumble F, Sinclair O (2022). Increasing uptake of maternal pertussis vaccinations through Funded Administration in Community pharmacies. Vaccines (Basel).

[51] Hawley S, Ali MS, Berencsi K, Judge A, Prieto-Alhambra D (2019). Sample size and power considerations for ordinary least squares interrupted time series analysis: a simulation study. Clin Epidemiol.

[52] Centers for Disease Control and Prevention. Guidelines for vaccinating pregnant women. https://www.cdc.gov/vaccines/pregnancy/hcp-toolkit/guidelines.html. 2023.

[53] Villar J, Soto Conti CP, Gunier RB, Ariff S, Craik R, Cavoretto PI (2023). INTERCOVID-2022 International Consortium. Pregnancy outcomes and vaccine effectiveness during the period of omicron as the variant of concern, INTERCOVID-2022: a multinational, observational study. Lancet.

