# Supplementary material for: Impact of national recommendations for routine pertussis vaccination during pregnancy on infant pertussis in Ontario, Canada: a population-based time-series study
Source: BMC Pregnancy Childbirth. 2023 Aug 31;23:627. doi: 10.1186/s12884-023-05938-2 (PMC10469528; doi:10.1186/s12884-023-05938-2)
Supplement: Supplementary file 1 — Supplementary Material 1 [file 12884_2023_5938_MOESM1_ESM.docx]

**Supplemental Figure 1: Cumulative sum of standardized ordinary least square residuals for monthly rates of incident infant pertussis in Ontario, Canada, January 2011 to February 2020**


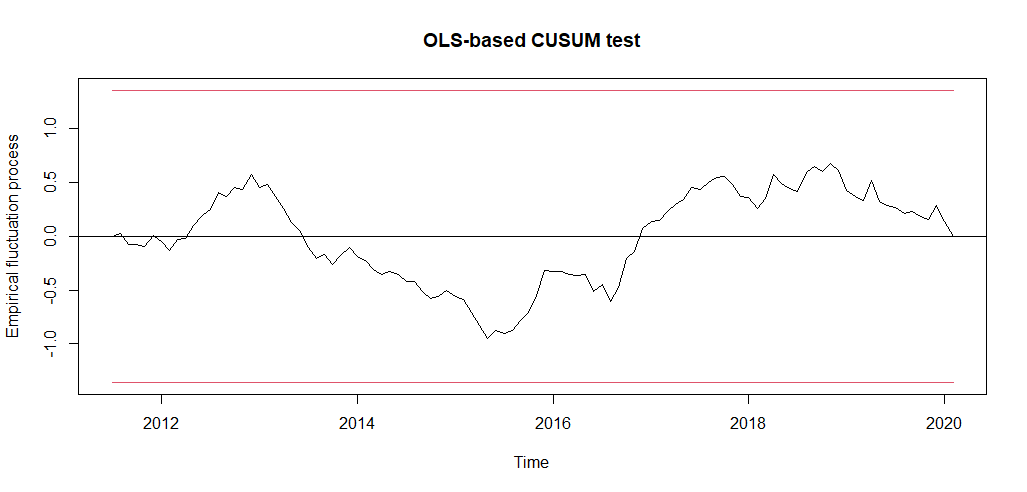


The time series data do not fall outside the OLS-CUSUM empirical fluctuation process test boundaries, indicating no structural change. The null hypothesis of parameter stability is not rejected at the 5% level because the CUSUM test statistic value of 0.87 is less than the 5% critical level of 1.36.
